# Engineered kirigami design of PVDF-Pt core–shell nanofiber network for flexible transparent electrode

**DOI:** 10.1038/s41598-023-29812-5

**Published:** 2023-02-14

**Authors:** Heesung Park, Hyeokjun Si, Junseo Gu, Donghyun Lee, Donghyuck Park, Young-In Lee, Kwanlae Kim

**Affiliations:** 1 Department of Manufacturing Systems and Design Engineering (MSDE), Seoul National University of Science and Technology (SeoulTech), Seoul, 01811 Republic of Korea; 2 Department of Materials Science and Engineering, Seoul National University of Science and Technology (Seoultech), Seoul, 01811 Republic of Korea

**Keywords:** Engineering, Materials science, Nanoscience and technology

## Abstract

Nanofiber networks comprising polymer-metal core–shell structures exhibit several advantages, such as high uniformities and considerable flexibilities. Additionally, the flexibility of the nanofiber network may be further enhanced by engineering the network topology. Therefore, in this study, the topologies of polyvinylidene fluoride (PVDF)-Pt core–shell nanofiber (CS NF) networks were engineered, and their performances as flexible transparent electrodes were comprehensively evaluated. Three distinct topologies of nanofiber networks were induced using circular, square, and rectangular electrode collectors. A highly uniform nanofiber network was obtained using the square electrode collector, which generated a high density of nanofiber junctions (nodes). Consequently, this nanofiber network exhibited the smallest sheet resistance $\left({R}_{\mathrm{s}}\right)$ and lowest optical transmittance $\left(T\right)$ among the three CS NF networks. In contrast, nanofiber bundles were frequently formed in the randomly aligned CS NF network prepared using the circular electrode collector, reducing the node density. As a result, it simultaneously exhibited a very small ${R}_{\mathrm{s}}$ and high $T$, generating the largest percolation figure of merit $\left(\Pi =330.5\right)$. Under certain strain directions, the CS NF network with the engineered topology exhibited a significantly enhanced mechanical durability. Finally, a flexible piezoelectric pressure sensor with CS NF network electrodes was fabricated and its sensing performance was excellent.

## Introduction

The advancement of flexible transparent electrode (FTE) technology has been fostered by demand for next-generation electronic devices^1,2^. Indium tin oxide (i.e. In_2_O_3_: Sn) is widely used as a TE because of its low sheet resistance $\left({R}_{\mathrm{s}}<80\Omega /\mathrm{sq}.\right)$ at a high optical transmittance $\left( {T = \sim 90 \% , \;550\; {\text{nm}}} \right)$^3,4^. However, it exhibits critical problems, such as a low abundance of indium and mechanical brittleness^5^. Accordingly, over the last decade, intensive research was conducted regarding nanostructured transparent conductors^6^. In these FTEs, nanomaterials, such as metal nanowires and carbon nanotubes, form conducting networks, resulting in excellent flexibilities, in addition to small ${R}_{\mathrm{s}}\left(\sim {10}^{2}-{10}^{3}\Omega /\mathrm{sq}.\right)$ values at high $T\left(\sim 90\%, 550 \mathrm{nm}\right)$ values^7,8^.

In these nanostructured transparent conductors, the percolation figure of merit $\left(\Pi \right)$ and exponent $(n)$ may be optimized by engineering the lengths^9^ and dispersion uniformities of 1D nanomaterials^10^. Nanofibers fabricated via electrospinning exhibit several advantages, such as high aspect ratios and uniformities, and are formed via facile fabrication processes^11^. Therefore, FTEs based on nanofiber networks were developed for various structures, e.g., a Cu nanofiber network exhibited a competitive ${R}_{\mathrm{s}}\left(50\Omega /\mathrm{sq}.\right)$ at a high *T* (90%, 550 nm)^12,13^. However, the performance of a metal nanofiber network as a TE is limited owing to the junction resistance between the nanofibers^14^. This constraint of the metal nanofiber network was overcome using a polymer nanofiber network as a template, upon which metal materials were directly deposited^15–17^. Due to the directionality of the thin film deposition processes, the deposited metal materials take a nanotrough structure. Subsequently, by dissolving the polymer template using an organic solvent or water, nanotrough networks were fabricated from the highly conductive metals such as Cu, Ag, Al and Au. Although this network is a competitive FTE in terms of a low ${R}_{\mathrm{s}}$ and high *T* (e.g., the ${R}_{\mathrm{s}}$ values of Cu, Au, and Ag at $\sim 90\mathrm{\%}$ *T* are $<10\Omega /\mathrm{sq}.$), the fabrication process is complicated^15^. From this perspective, a polymer-metal core–shell nanofiber (CS NF) network displays several advantages^18–24^. The fabrication process, which comprises only two steps (electrospinning and thin-film deposition), is applicable to numerous highly conductive materials. Furthermore, under tensile strain, the metal film deposited on a soft polymer forms multiple necks, delaying rupture and enhancing the mechanical durability of the metal film conductor^18,25,26^.

Generally, the flexibilities of stiff materials may be drastically improved by employing serpentine or network structures^27,28^. Nanofiber networks intrinsically adopt Kirigami designs, providing considerable structural flexibilities. Furthermore, when a nanofiber network exhibits a serpentine structure, the flexibility of the electrode is further enhanced, e.g., the mechanical durabilities of a polyvinylpyrrolidone(PVP)-Au CS NF network and an Au nanotrough network were considerably enhanced under stretching by employing buckled nanofibers^29,30^. Additionally, the stretchability of the network structure is highly influenced by topology, e.g., the stretchabilities of Au nanomesh electrodes were considerably enhanced (e.g., no noticeable fatigue after repetitive stretching to > 100% strain) via a theoretical approach in designing the optimal topology^31^. Similarly, the flexibility of the nanofiber network may be further enhanced by engineering the network topology. In this case, the nanofiber network fabricated via electrospinning is advantageous because the alignment of nanofibers may be controlled by designing the pattern of the electrode collector^32–34^.

In this study, three distinct topologies of polyvinylidene fluoride (PVDF) nanofiber networks were prepared using circular, square, and rectangular electrode collectors. PVDF-Pt CS NF networks were fabricated by coating the PVDF nanofibers with Pt. The $\Pi$ values of the three distinct fiber networks were evaluated by measuring ${R}_{\mathrm{s}}$ and *T*. The flexibilities of the nanofiber networks were assessed via bending and stretching studies. Finally, the PVDF-Pt CS NF network electrodes were applied in flexible piezoelectric pressure sensors to demonstrate their performances as electrodes. To the best of our knowledge, a polymer-metal CS NF network has not been used as the electrodes of piezoelectric pressure sensors, in which strong electric field should be applied through the electrodes for an electrical poling process. Flexible and transparent pressure sensors can be applied to electronic skins and wearable self-powered touch sensors in which excellent transparency and flexibility are simultaneously required to enhance wearing sensation, aesthetics, and security in military applications^35,36^.

## Methods

### Fabrication of the PVDF-Pt CS NF networks

**Figure 1:**
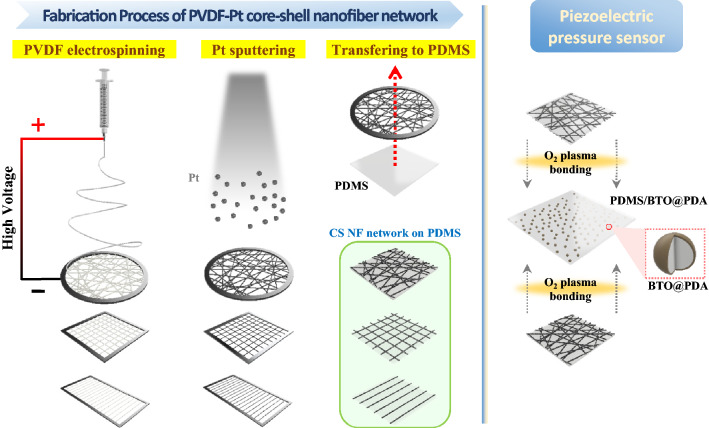
Schematic diagram of the fabrication processes of the CS NF networks, comprising PVDF electrospinning, Pt sputtering, and transferring to a PDMS substrate. The piezoelectric composite comprising a PDMS matrix and BTO@PDA nanoparticles is sandwiched by the top and bottom CS NF networks on PDMS substrates to fabricate a piezoelectric pressure sensor.

The PVDF nanofiber was used as the core material because of its high flexibility^37^. PVDF powder (1.5 g, molecular weight ≈ 534 000 g/mol, Sigma Aldrich, St. Louis, MO, USA) was dissolved in 8.5 g of the solvent via stirring at 100 °C for 1 h to prepare a PVDF solution with a concentration of 15 wt.%. The solvent consisted of 7 mL acetone (Duksan Reagents, Ansan, Republic of Korea) and 3 mL dimethylformamide (Duksan Reagents). The PVDF nanofibers were produced using an electrospinning/-spray system (ESR200, NanoNC, Seoul, Republic of Korea). In this study, fabricating a free-standing PVDF nanofiber network was essential in ensuring that the nanofibers could be conformally coated with Pt during sputtering. Free-standing PVDF nanofiber networks may be produced under specific voltage and tip-to-collector distance (TCD) conditions^38^. In this study, a TCD, voltage, and solution flow rate of 17 cm, 10 kV, and 40 μL/min, respectively, were consistently used during electrospinning. As shown in Fig. 1, circular (0.021 m radius), square (0.042 × 0.042 m), and rectangular (0.11 × 0.042 m) electrode collectors were used to control the topologies of the PVDF nanofiber networks. The electrode collectors, which were comprised of stainless steel, were placed 2 cm above the floor to prevent the PVDF nanofibers from adhering to the floor.

Pt was used as an electrode material due to its high electrical conductivity and resistance to oxidation and corrosion^39^. Nevertheless, depending on the specific applications of electrode materials, other cost-effective metals may be also considered as an electrode material. Prior to deposition of the Pt layer, the PVDF nanofibers were heated (80 °C for 2 h) in an oven to remove the residual solvent. The PVDF nanofibers were coated with Pt via direct-current magnetron sputtering (KVS-2000, Korea Vacuum Tech, Gimpo, Republic of Korea) with a Pt sputter target (99.999% purity, RND Korea, Gwangmyeong, Republic of Korea). Sputtering was used as the thin-film deposition technique because sputtered atoms are scattered by the inert gas in the chamber, resulting in a wide range of incidence angles to the PVDF nanofibers. Additionally, sputtering does not typically require a high-temperature environment, and thus, it is suitable for use in coating metals onto PVDF nanofibers. Sputtering was performed at base and working pressures of $2\times {10}^{-5} \mathrm{Torr}$ and 15 mTorr, respectively, and a power of 150 W. Finally, the resultant CS NF networks were transferred to polydimethylsiloxane (PDMS) substrates for use in fabricating the piezoelectric pressure sensors.

### Fabrication of the piezoelectric pressure sensors

BaTiO_3_ (BTO) nanoparticles were functionalized with polydopamine (PDA)^40,41^, as described in the Supplementary Information. BTO@PDA nanoparticles (6 g) were ultrasonically dispersed in 14 g ethanol (Duksan Reagents) for 1 h. Subsequently, 14 g of PDMS resin (Sylgard 184 Silicone Elastomer Kit, Dow Corning, Midland, MI, USA) was added to the solution and dispersed for 30 min. The solution was stirred at 60 °C at 300 rpm for 24 h to evaporate the residual ethanol. Crosslinker (1.4 g, Sylgard 184 Silicone Elastomer Kit, Dow Corning) was then added to cure the solution. Finally, a PDMS/BTO@PDA composite layer was prepared via spin coating at 400 rpm for 1 min and annealing at 100 °C for 2 h in an oven.

As shown in Fig. 1, the prepared PDMS/BTO@PDA composite layer is bonded to the CS NF network electrodes on PDMS substrates. During this process, the contact surfaces of the electrodes and PDMS/BTO@PDA layer are treated with oxygen plasma to enhance chemical adhesion during bonding^42^. Subsequently, the PDMS/BTO@PDA composite layer sandwiched between the top and bottom electrodes is annealed at 60 °C for 35 min in an oven. Finally, DC electrical poling was conducted on the PDMS/BTO@PDA composite through the CS NF networks at an electric field intensity of 30 MV/m for 1 h at room temperature.

### Characterizations

The fabricated CS NF networks were transferred to PDMS substrates for use in conductive atomic force microscopy (C-AFM), *T* measurements, and mechanical durability studies. Slide glass and Si wafers were used as substrates in ${R}_{\mathrm{s}}$ measurements and energy dispersive X-ray spectroscopy (EDS), respectively. The topologies of the nanofiber networks were studied using scanning electron microscopy (SEM, JSM-6700F, JEOL, Tokyo, Japan), which was also used to analyze the surface quality of the Pt layer and cross-section of the PVDF-Pt CS NF. The orientations of the PVDF nanofibers in these nanofiber networks were analyzed using ImageJ software (National Institutes of Health, Bethesda, MD, USA) in conjunction with the SEM images. More specifically, OrientationJ, which is an ImageJ plug-in, was used to statistically analyze the orientation of PVDF nanofibers. EDS (SU8010, Hitachi, Tokyo, Japan) was used to conduct elemental mapping of the PVDF-Pt CS NFs, and their electrical conductivities were measured using C-AFM. A C-AFM module and current amplifier were installed in an AFM (XE-150, Park Systems, Suwon, Republic of Korea)^43^, and a probe (ElectriMulti75-G, BudgetSensors, Sofia, Bulgaria) with a spring constant of 3 $\mathrm{N}/\mathrm{m}$ was used to ensure good electrical contact between the AFM probe and the nanofibers. AFM measurements were typically conducted at a set point and scan rate of 30 nN and 0.5 Hz, respectively, with a tip bias of 5 mV applied during C-AFM.

The ${R}_{\mathrm{s}}$ of the CS NF network was measured using the four-point probe method (M4P-302, MSTECH, Hwaseong, Republic of Korea) interfaced with a source measure unit (2612B, Keithley Instruments, Cleveland, OH, USA). Optical analysis was conducted by an ultraviolet–visible spectrophotometer (Specord 200 PLUS, Analytik Jena, Jena, Germany) and a haze meter (NDH 5000, Nippon Denshoku, Japan). To measure the changes in the resistances of the CS NF networks induced by mechanical strain, the resistance between the two ends of the rectangular sample was monitored. A bending tester (JIBT-200, Junil Tech, Republic of Korea) interfaced with a source meter was used to evaluate the mechanical durability of the CS NF network.

Transmission electron microscopy (TEM, JEM-2010, JEOL) was used to observe the thicknesses of the PDA layers of the BTO@PDA nanoparticles. X-ray photoelectron spectroscopy (XPS, K-Alpha+, Thermo Fisher Scientific, Waltham, MA, USA) was used to determine the chemical compositions of the PDA layers, and the XP spectra were analyzed using a fitting program (Avantage Data System (Thermo Fisher Scientific) and CasaXPS (Casa Software, Teignmouth, UK)). The dielectric properties of the PDMS/BTO@PDA composites were studied using an LCR meter (E4980AL, Keysight Technologies, Santa Rosa, CA, USA). Finally, the leakage current of the composite was measured using a ferroelectric measurement system (TF 1000, aixACCT Systems, Aachen, Germany) interfaced with a high-voltage amplifier (Trek 10/10B-HS, Advanced Energy, Denver, CO, USA).

## Results and discussion

**Figure 2:**
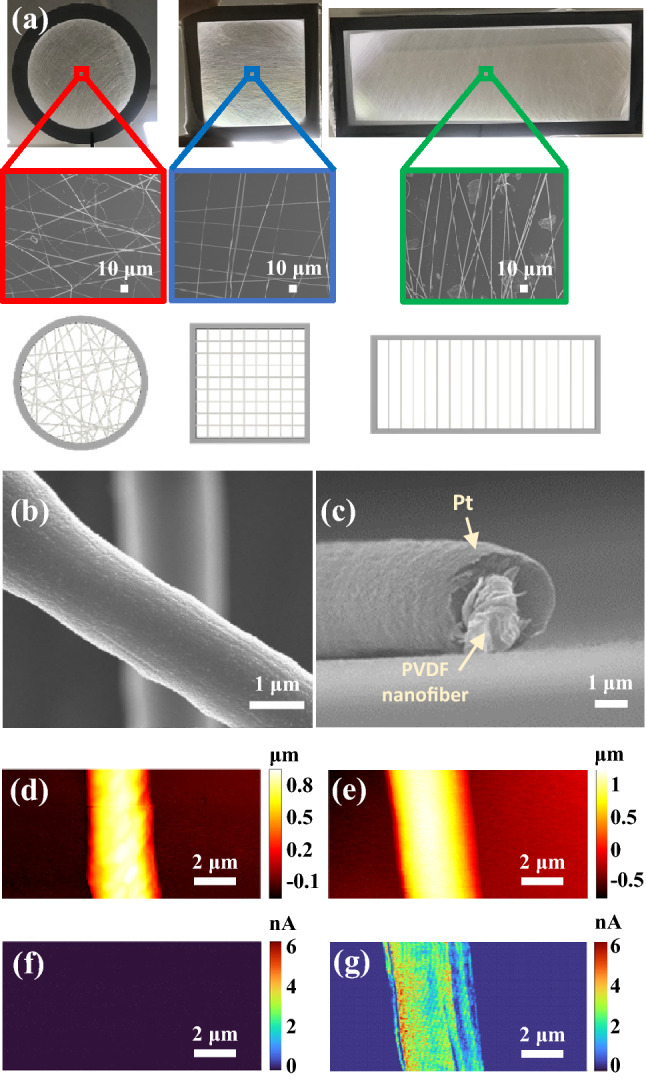
Topologies of the CS NF networks and characterization of a Pt-coated PVDF nanofiber. (**a**) PVDF nanofibers formed using the circular, square, and rectangular electrode collectors, and the magnified images of the nanofiber networks. The schematic diagrams show the simplified topologies of the nanofiber networks. SEM images of the (**b**) surface and (**c**) cross-section of a Pt-coated PVDF nanofiber, AFM topography images of a PVDF nanofiber (**d**) before and (**e**) after Pt sputtering, and C-AFM images of a PVDF nanofiber (f) before and (**g**) after Pt sputtering. The Pt sputtering time used for (**b**), (**c**), (**e**), and (**g**) was 4 min.

The advantage of the nanostructured conductor based on electrospun nanofibers lies in the controllable topology of the nanofiber network according to the shape of electrode collector^34^. Figure 2a shows the three types of electrode collectors and PVDF nanofibers formed via electrospinning. Magnified images of the nanofibers formed using the circular, square, and rectangular electrode collectors are shown in Fig. 2a. The orientations of the PVDF nanofibers in these nanofiber networks were analyzed using OrientationJ. This program computes a structure tensor for each pixel and is often used to identify the local orientation of each pixel of the image^44,45^. For the present study, the size of local window $\left(\sigma \right)$ was set as 1 pixel, and the Gaussian method was used to numerically estimate gradients. The minimum energy of 1% was applied to ignore the background noise originating from the substrate. OrientationJ has been often used to analyze the orientation of nanofibers^46,47^. This methodology was applied to twelve CS NF network samples. As shown in Supplementary Fig. S1, whereas no distinct peak is consistently observed for the nanofiber alignment obtained using the circular electrode collector, strong peaks may be clearly and consistently observed for those obtained using the square and rectangular electrode collectors. As shown in Supplementary Fig. S1b, the peaks near 0° and ± 90° indicate that most PVDF nanofibers are closely oriented in the horizontal and vertical directions, respectively. Conversely, as shown in Supplementary Fig. S1c, the peak near ± 90° represents vertically oriented nanofibers. Notably, using the square and rectangular electrode collectors, numerous nanofibers are slightly deviated from the horizontal and vertical orientations. Additionally, as Supplementary Fig. S1d and e show, the average distance between neighboring nanofibers and the area fraction of nanofibers slightly varied from sample to sample. See the Supplementary Information for further details. Based on this result and the SEM images shown in Fig. 2a, the simplified topologies of the PVDF nanofibers may be shown by the schematic diagrams at the bottom of Fig. 2a. Meanwhile, using the circular electrode collector, numerous nanofibers coalesce during electrospinning, generating nanofiber bundles. Conversely, using the square electrode collector, the orientation of the PVDF nanofiber alignment is frequently changed from vertical to lateral or vice versa. This is because the electrostatic force acting on an electrified liquid jet and the square electrode collector has no preferential orientation between the two pairs of parallel electrodes of the square electrode collector^33^. This frequently switching orientation of the nanofiber alignment prevented the formation of nanofiber bundles, providing more uniformity in the nanofiber network. Supplementary Fig. S2 shows high-magnification SEM images of the PVDF nanofibers formed using the circular and square electrode collectors to reveal this difference.

The surface of a Pt-coated PVDF nanofiber (4 min of sputtering) is shown in Fig. 2b. The sputtering conditions described in the Methods section enable the conformal coating by the Pt layer. Furthermore, Supplementary Fig. S3 shows that the junctions of the PVDF nanofibers are fused by the Pt layers, reducing the contact resistance between the nanofibers^15,20^. The core–shell structure may be clearly identified based on the cross-sectional view of the Pt-coated PVDF nanofiber shown in Fig. 2c. AFM and C-AFM were used to measure the diameters and electrical conductivities of the Pt-coated PVDF nanofibers, respectively. Figure 2d and e show the respective topographic images of a single PVDF nanofiber before and after Pt sputtering for 4 min. The measured diameter of the Pt-coated PVDF nanofiber shown in Fig. 2e is 2.72 μm, which is larger than that (1.71 μm) observed in the SEM image shown in Fig. 2b. This error is due to the finite response time of the AFM feedback system and the physical contact between the PVDF nanofiber and the sidewall of the AFM tip. In particular, this tip-related artefact occurs when a large object is imaged by an AFM tip^48–50^. The size of PVDF nanofibers measured by AFM should vary depending on the size of the AFM tip. After Pt sputtering, the nanofiber diameter increases from 2.59 to 2.72 μm, with a growth rate of 32.5 $\mathrm{nm}/\mathrm{min}$, as shown in Fig. 2d and e. The deposition of the Pt layer on the PVDF nanofiber is confirmed by the C-AFM images. Compared with the C-AFM image captured before Pt sputtering (Fig. 2f), strong current signals $\left( { \le 6.12\;{\text{nA}}} \right)$ are detected in the Pt-coated PVDF nanofiber (Fig. 2g). The presence of PVDF-Pt CS NFs is also indicated by the results of elemental mapping (Supplementary Fig. S4). The diameters of the PVDF nanofibers obtained using each electrode collector were statistically analyzed (Supplementary Fig. S5). The range of the average diameter is only 1.524–1.568 μm, depending on the shape of the electrode collector. Therefore, the effect of nanofiber size on the properties of the CS NF networks is not considered in this study.

**Figure 3:**
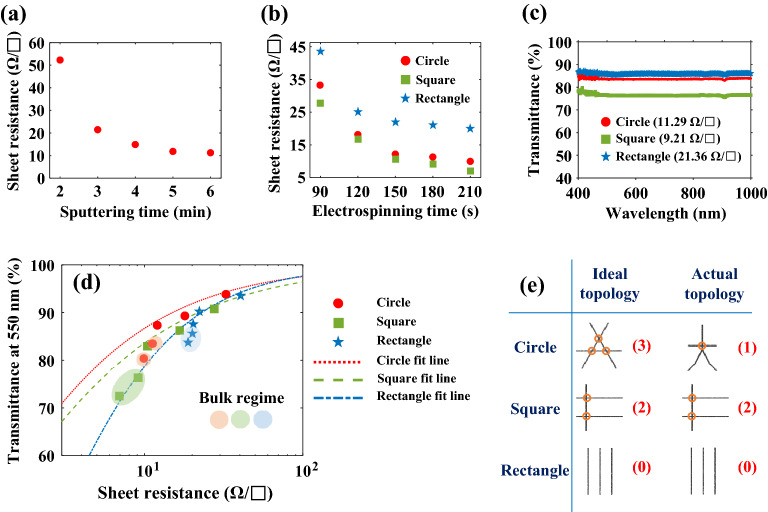
Sheet resistances (${R}_{\mathrm{s}}$) and transmittances (*T*) of the CS NF networks obtained using the circular, square, and rectangular electrode collectors. (**a**) ${R}_{\mathrm{s}}$ of the CS NF network obtained using the circular electrode collector with respect to sputtering time, (**b**) ${R}_{\mathrm{s}}$ values of the CS NF networks with respect to electrospinning time, (**c**) *T* values of the CS NF networks (180 s electrospinning time) with respect to wavelength, and (**d**) *T* values (at 550 nm) of the CS NF networks with respect to ${R}_{\mathrm{s}}$. (**e**) Number of nodes depending on the ideal and actual topologies.

Subsequently, to evaluate the performances of the CS NF networks as FTEs, their ${R}_{\mathrm{s}}$ and *T* values were assessed. The ${R}_{\mathrm{s}}$ values of the CS NF networks depend strongly on their sputtering and electrospinning times. As shown in Fig. 3a, the ${R}_{\mathrm{s}}$ of the CS NF network obtained using the circular electrode collector was measured at various sputtering times from 2 to 6 min. When the sputtering time is 6 min, ${R}_{\mathrm{s}}$ decreases to approximately 11.29 $\Omega /\mathrm{sq}$., which is sufficiently low compared to those reported in the previous studies of FTEs based on nanofibers^14,17,21,51^. Therefore, a sputtering time of 6 min was consistently used during the fabrication of the CS NF networks. Thereafter, the ${R}_{\mathrm{s}}$ values of the CS NF networks were measured while gradually increasing the electrospinning time from 90 to 210 s. Notably, for the CS NF networks prepared using the square and rectangular electrode collectors, ${R}_{\mathrm{s}}$ depends on measurement direction, as shown in Supplementary Fig. S6. Thus, their average values are used in Fig. 3b. The CS NF network obtained using the square electrode collector exhibits the smallest ${R}_{\mathrm{s}}$ followed by those of the CS NF networks obtained using the circular and rectangular electrode collectors (e.g., at an electrospinning time of 210 s, $R_{{\text{s}}} \left( {{\text{square}}} \right) = 6.97\;{\Omega }/{\text{sq}}.$, $R_{{\text{s}}} \left( {{\text{circle}}} \right) = 9.94\;{\Omega }/{\text{sq}}.$, and $R_{{\text{s}}} \left( {{\text{rectangle}}} \right) = 18.88\;{\Omega }/{\text{sq}}.$).

Figure 3c shows the *T* values of the CS NF networks (electrospinning and sputtering times of 180 s and 6 min, respectively). The *T* values of the nanofiber networks decrease as the electrospinning time increases, regardless of the type of electrode collector used (Supplementary Fig. S7a–c). The relationship between transparency and electrospinning time may also be confirmed by directly comparing the images of the CS NF networks prepared at various electrospinning times (1.5–3.5 min, Supplementary Fig. S7d). Finally, the measured haze values of CS NF networks with respect to ${R}_{\mathrm{s}}$ are shown in Supplementary Fig. S7e. In nanostructured conductors, haze values are dependent on network densities^52,53^. The measured haze of the CS NF network ranged from 3.65 to 11.81% depending on electrospinning times (1.5–3.5 min), and the topologies of nanofiber networks. Among the CS NF network with 1.5 min electrospinning time, the smallest haze was measured from the CS NF network from the rectangular electrode collector (3.65%), followed by circular (5.02%) and square electrode collector (7.67%).

For the CS NF networks prepared using electrospinning times of 90–210 s, *T* at 550 nm wavelength with respect to ${R}_{\mathrm{s}}$ is shown in Fig. 3d. For each electrode collector, the plot may be divided into the percolation and bulk regimes^7,8^. As shown in Fig. 3d, the data points representing the bulk regime are shaded using ellipses and circles. Only the computed fit lines of the CS NF networks in the percolation regime are shown. The relationship between ${R}_{\mathrm{s}}$ and *T* in the percolation regime is typically described by^7,8^

1 $$T = \left[ {1 + \frac{1}{{\Pi }}\left( {\frac{{Z_{0} }}{{R_{s} }}} \right)^{{1/\left( {n + 1} \right)}} } \right]^{ - 2} ,$$

where $\Pi$, *n*, and ${Z}_{0}$ are the percolation figure of merit and percolation exponent, and impedance of free space (377 $\Omega$), respectively. The relationship between ${R}_{\mathrm{s}}$ and *T* in the percolation regime is clear in the plot of $\mathrm{Log}\left({T}^{-0.5}-1\right)$ as a function of $\mathrm{Log}\left({R}_{\mathrm{s}}/{Z}_{0}\right)$ (Supplementary Fig. S8). The $\Pi$ and *n* values of the CS NF networks obtained using the circular, square, and rectangular electrode collectors were computed by applying the data shown in Fig. 3d to Eq. (1). The obtained $\Pi$ and *n* data are shown in Table 1. Generally, excellent transparent conductors with small ${R}_{\mathrm{s}}$ and large *T* values display large $\Pi$ and small *n* values^7,8^. The CS NF network obtained using the circular electrode collector exhibits the largest $\Pi$ (330.5) and smallest *n* (0.026) compared to those of the CS NF networks obtained using the other electrode collectors ($\Pi \left(\mathrm{square}\right)=198.6$, $\Pi \left(\mathrm{rectangle}\right)=168.2$, $n\left(\mathrm{square}\right)=0.135$, and $n\left(\mathrm{rectangle}\right)=0.238$). The CS NF network obtained using the circular electrode collector simultaneously displays a very small ${R}_{\mathrm{s}}$ and high *T* compared to those of the other networks, as shown in Fig. 3b and c. Notably, the $\Pi$ and *n* values of the CS NF networks fabricated in this study are outstanding compared to those of previously reported nanostructured transparent conductors (Table 1). This is attributed to three factors: the high uniformities of the electrospun nanofibers, the conformal coating of Pt via sputtering, and the low junction resistances.

**Table 1:** Percolation exponents (*n*) and figures of merit $\left(\Pi \right)$ of the CS NF networks fabricated in this study and those of the nanostructured transparent conductors reported in previous studies^12,15,18,52,54–61^.

Nanostructured transparent conductors	*n*	$\Pi$	References
PVDF-Pt core–shell nanofiber (Circle)	0.026	330.5	Present work
PVDF-Pt core–shell nanofiber (Square)	0.135	198.6	Present work
PVDF-Pt core–shell nanofiber (Rectangle)	0.238	168.18	Present work
Ag nanowires	1.9	31.7	^54^
Graphene	3.1	3.5	^55^
Cu nanofibers	0.43	75.37	^12^
Al nanotrough	0.33	48	^15^
Ag nanotrough	0.10	495	^15^
Au nanotrough	0.07	1030	^15^
Dip-coated long Ag nanowires	0.65	162.54	^56^
Dip-coated short Ag nanowires	1.62	58.04	^56^
Cell shaped Ag nanowires	1.48	46.43	^57^
Ag metallic network	1.58	54.64	^58^
Patterned Ag nanowires	0.13	291.61	^59^
Aligned Ag nanowires micro-grids	0.24	575.72	^60^
Epoxy-embedded Ag nanowires	1.19	52	^61^
Ag nanowire-SWCNT hybrid	0.37	245.8	^52^
Au coated nanofier	0.255	78.135	^18^

In comparison with those reported in earlier studies, the PVDF-Pt CS NF network prepared using the circular electrode collector exhibits an outstanding performance as a transparent conductor because of its large percolation figure of merit $\left(\Pi =330.5\right)$ and small percolation exponent $\left(n=0.026\right)$.

The effect of nanofiber network topology on ${R}_{\mathrm{s}}$ and *T* may be theoretically explained using node theory^62^. A large number of nodes (nanofiber junctions) facilitates charge transport but simultaneously reduces *T*. As shown in the schematic diagram (Fig. 3e), the number of nodes depends strongly on the type of electrode collector used. When three nanofibers are uniformly dispersed using each type of collector, the numbers of nodes are 3 (circles), 2 (squares), and 0 (rectangles). However, nanofiber bundles are frequently produced using the circular electrode collector, reducing the number of nodes to 1 (circle). Therefore, according to node theory, based on the actual topology, the smallest ${R}_{\mathrm{s}}$ may be expected upon using the square electrode collector, followed by those of the networks obtained using the circular and rectangular electrode collectors. In contrast, the highest *T* may be expected upon using the rectangular electrode collector, followed by those of the networks obtained using the circular and square electrode collectors. The experimental results shown in Fig. 3b and c may be clearly explained by node theory, based on the actual topology. It should be noted that there are actually a number of nodes in the nanofiber network from the rectangular electrode collector as the SEM image in Fig. 2a shows. Nodes can be generated by neighboring nanofibers unless nanofibers are perfectly parallel to each other. For this reason, the CS NF network from the rectangular electrode collector can be still electrically conductive along the nanofiber orientation. However, in general a node cannot be generated by the nanofibers far apart.

Meanwhile, the uniformity of ${R}_{\mathrm{s}}$ and $T$ over the entire CS NF network generated within the electrode collector was also evaluated. In the present study, three square samples $\left(1\mathrm{ cm }\times 1\mathrm{ cm}\right)$ were taken from the locations shown in Supplementary Fig. S9c, and the measured ${R}_{\mathrm{s}}$ and $T$ values are represented in Supplementary Fig. S9a and b, respectively. Note that ${R}_{\mathrm{s}}$ was measured from five different locations in each sample, and their averaged values were used in Supplementary Fig. S9a. For the CS NF networks from the square and rectangular electrode collectors, there were considerable differences in ${R}_{\mathrm{s}}$ and *T* between location 3 and the rest due to the non-uniform nanofiber alignments at the corners of the square and rectangular electrode collector. Therefore, in the present study all the CS NF network samples were taken from the central area of the electrode collectors for the characterizations and device fabrication. For the large-area fabrication of the nanofiber networks, the electrode collector with a larger diameter or side length may be used adjusting TCD and electrospinning voltage. In this way, the area of the nanofiber network with good uniformity can be also enlarged. Subsequently, the mechanical durabilities of the CS NF networks under mechanical stress were assessed via bending and stretching studies. First, the excellent flexibility of a CS NF network was compared to that of a Pt thin film via a bending study at a bending radius of 1 mm over 1000 cycles (Supplementary Fig. S10). Whereas microcracks may be observed in several regions of the Pt thin film, the CS NF network is well preserved under bending motions for two reasons. First, the strain induced by bending is alleviated by the rearrangement of the nanofibers within the network. Second, the rupturing of the CS NF network under severe bending is avoided due to the small diameters of the flexible PVDF nanofibers^12,18^.

**Figure 4:**
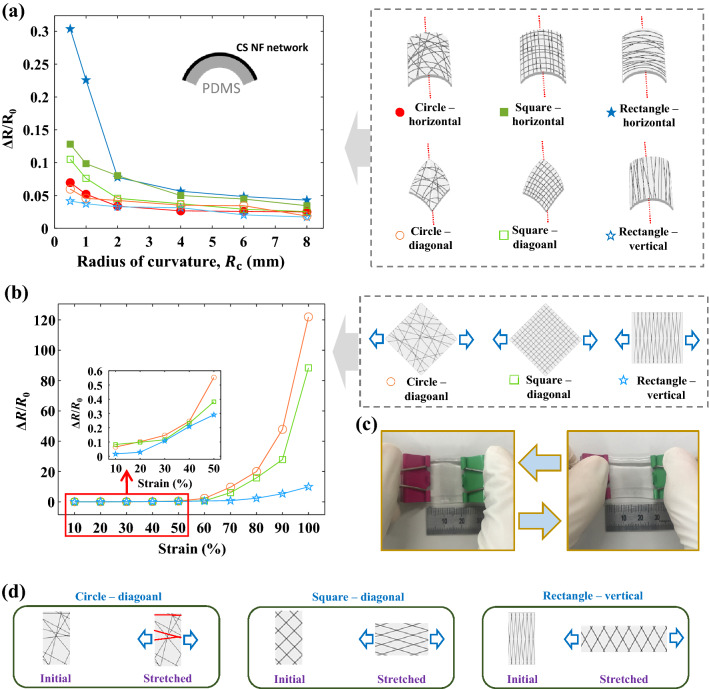
Mechanical durability evaluations of the CS NF networks obtained using the circular, square, and rectangular electrode collectors. (**a**) $\Delta R/{R}_{0}$ values of the CS NF networks after 1000 cycles of bending and releasing at various bending radii (the radii of curvature, ${R}_{\mathrm{c}}$). The bending directions with respect to the topologies of the nanofiber networks are shown in the schematic diagrams. (**b**) $\Delta R/{R}_{0}$ values of the CS NF networks after 10 cycles of stretching and releasing at various strains. The stretching directions with respect to the topologies of the nanofiber networks are shown in the schematic diagrams. (**c**) Images showing the stretching and releasing of a CS NF network on a PDMS substrate. (**d**) Schematic diagrams showing the rearrangements of PVDF nanofibers under stretching. The PVDF nanofibers shown in red are highly strained under tensile stress.

More systematic bending studies were performed using the three CS NF networks. As shown in Fig. 4a, the $\left(R-{R}_{0}\right)/{R}_{0}$ values of the CS NF networks were measured after 1000 cycles of bending and releasing, gradually reducing the bending radius from 8 to 0.5 mm. ${R}_{0}$ and $R$ are the resistances before and after the mechanical durability studies, respectively. During the bending studies, tensile stress was induced in the CS NF network by placing it on the outer surface of a bent PDMS substrate. The tensile strain induced during the bending study may be computed as follows^51,63^:

2 $${\text{Strain}} = \frac{{d_{{{\text{substrate}}}} + d_{{{\text{network}}}} }}{{2 \times R_{{\text{c}}} }},$$

where $R_{{\text{c}}}$ is the radius of curvature and $d_{{{\text{substrate}}}}$ (= 140 μm) and $d_{{{\text{network}}}}$ (= 2 μm) are the thicknesses of the PDMS substrate and CS NF network, respectively. The highest strain (14.2%) is induced when $R_{{\text{c}}} = 0.5{\text{ mm}}$. The effect of the bending orientation on $\Delta R/R_{0}$ of the CS NF network was also investigated. The bending orientations of each CS NF network are shown in Fig. 4a. The CS NF network obtained using the rectangular electrode collector exhibits the optimal and worst mechanical durabilities, depending on the bending orientation. When most nanofibers are aligned with the direction of tensile strain (corresponding to “Rectangle–horizontal”), they are subjected to severe strain, resulting in the largest $\Delta R/R_{0}$ (0.304 at $R_{{\text{c}}} = 0.5\;{\text{mm}}$). In contrast, when most of the nanofibers are oriented perpendicular to the direction of tensile strain, the smallest $\Delta R/R_{0}$ (0.042 at $R_{{\text{c}}} = 0.5\;{\text{mm}}$) is observed. In the case of the CS NF network obtained using the square electrode collector, the $\Delta R/R_{0}$ of “Square–horizontal” (0.128 at $R_{{\text{c}}} = 0.5\;{\text{mm}}$) is larger than that of “Square–diagonal” (0.116 at $R_{{\text{c}}} = 0.5 {\text{mm}}$), as approximately half of the nanofibers in the “Square–horizontal” orientation are closely aligned with the direction of tensile strain. Notably, the CS NF networks display excellent mechanical durabilities under repetitive bending with $\Delta R/R_{0} \le 0.304$ at $R_{{\text{c}}} = 0.5\;{\text{mm}}$, regardless of bending orientation.

To apply the CS NF network in wearable electronic devices, an excellent mechanical durability under stretching is required. Thus, $\Delta R/{R}_{0}$ was measured after 10 cycles of stretching and releasing (Fig. 4c), and the tensile strain was gradually increased from 10 to 100% (Fig. 4b). Notably, the stretching study was performed in the orientations “Circle–diagonal”, “Square–diagonal”, and “Rectangle–vertical”, as these orientations exhibit superior flexibilities during the bending studies. As shown in the inset of Fig. 4b, the CS NF networks exhibit $\Delta R/{R}_{0}<0.55$ at strains of ≤ 50%, confirming their excellent stretchabilities. At 10% strain, the largest $\Delta R/{R}_{0}$ is observed for “Square–diagonal” (0.08), followed by those of “Circle–diagonal” (0.06) and “Rectangle–vertical” (0.02), because of the small ${R}_{0}$ of “Square–diagonal” (this is consistent with the results of ${R}_{\mathrm{s}}$ measurement shown in Fig. 3b). These results display the same trend as that of the results of the bending study shown in Fig. 4a. However, when the strain is > 20%, “Circle–diagonal” exhibits the largest $\Delta R/{R}_{0}$, and at a strain of 100%, $\Delta R/{R}_{0}\left(\mathrm{rectangle}\right)=9.93$, $\Delta R/{R}_{0}\left(\mathrm{square}\right)=88.37$, and $\Delta R/{R}_{0}\left(\mathrm{circle}\right)=121.96$.

When a CS NF network is subjected to tensile stress, the structure accommodates the resultant strain via topological transition. During the rearrangement of the nanofibers, brittle Pt thin films may maintain their conductivities by forming multiple necks, preventing macroscopic rupture^18^. When the stretched nanofiber network is subjected to a larger tensile stress, a noticeable increase in resistance occurs, possibly due to the destruction of the fused junctions and the disconnection at the central region of the nanofibers^17^. The schematic diagrams shown in Fig. 4d display the different rearrangement processes of the nanofibers based on the topology of the nanofiber network. Within “Circle–diagonal”, the rearrangement of the nanofibers is constrained, and several nanofibers (shown in red) closely aligned with the stretching direction should be highly strained. In contrast, in “Square–diagonal” and “Rectangle–vertical”, almost no nanofibers are closely aligned with the stretching directions. Among these three cases, the largest $\Delta R/{R}_{0}$ of “Circle–diagonal” may be explained by this interpretation. Additionally, for the given stretching motion, the topology of “Rectangle–vertical” is more favorable for the rearrangement of the nanofibers than that of “Square–diagonal”. Therefore, the largest degree of deformation without the destruction of the fused nanofibers should be observed in the topology of “Rectangle–vertical”, followed by those of the topologies of “Square–diagonal” and “Circle–diagonal”. The results of the stretching study shown in Fig. 4b are consistent with this theoretical interpretation. Given the stretchability of the skin ($\sim 30{\text{\% }}$), these CS NF networks exhibit excellent stretchabilities for use in the fields of wearable electronics and soft robotics^28^.

CS NF networks were also placed on the inner surfaces of the bent PDMS substrates to induce compressive strain, and the $\Delta R/{R}_{0}$ values measured after 1000 bending cycles at ${R}_{\mathrm{c}}=1 \mathrm{mm}$ are shown in Supplementary Fig. S11a. Clearly, $\Delta R/{R}_{0}$ under compressive strain is much smaller than that under tensile strain (Supplementary Fig. S11b). “Rectangle–horizontal” is the most fragile case under compressive and tensile strains. Finally, to assess the durability of CS NF networks under harsh conditions, $\Delta R/{R}_{0}$ was monitored during scotch tape peel-off, and sonication tests. The experimental method is introduced in Supplementary Fig. S12. As Supplementary Fig. S12a and d show, there was no considerable increase in $\Delta R/{R}_{0}$ of the CS NF networks under such harsh conditions.

**Figure 5:**
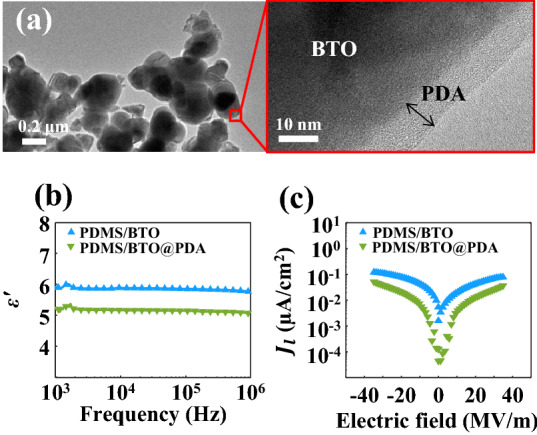
Characterizations of the BTO@PDA and PDMS/BTO@PDA composites. (**a**) TEM images of BTO@PDA, including a magnified view of the PDA layer, and the (**b**) dielectric permittivities $\left( {\varepsilon^{\prime}} \right)$ and (**c**) leakage current densities $\left( {J_{l} } \right)$ of the PDMS/BTO and PDMS/BTO@PDA composites with BTO concentrations of 20 wt.%.

To assess the performance of the CS NF network as a flexible electrode, a piezoelectric pressure sensor was fabricated (Fig. 1 shows the device structure). It is noted that in the present work the PVDF-Pt CS NF network from the circular electrode collector was used to fabricate a piezoelectric pressure sensor as its $\Pi$ value was the largest among the three CS NF networks. However, when greater conductivity or stretchability is required, the other CS NF networks may be also applied to the pressure sensor. The piezoelectric composite comprises a PDMS matrix and BTO nanoparticles, which are functionalized using PDA to aid the dispersion of BTO and reduce leakage current during electrical poling^64,65^. The PDA layer with a thickness of $\sim \;9{\text{ nm}}$ may be clearly observed in the enlarged TEM image shown in Fig. 5a. The presence of the PDA layer may also be verified using XPS, as shown in Supplementary Fig. S13. The effect of the PDA coating on the electrical properties of the PDMS/BTO@PDA composite was studied by measuring the dielectric permittivity $\left( {\varepsilon^{\prime}} \right)$ of the composite. As shown in Fig. 5b, the $\varepsilon^{\prime}$ of the PDMS/BTO composite with a BTO concentration of 20 wt.% decreases with the formation of the PDA layers on the BTO nanoparticles in the frequency range of 10^3^–10^6^ Hz. This is attributed to the decreased interfacial polarization caused by the improved interfacial compatibility between the PDMS matrix and BTO nanoparticles^64,65^. Due to the reduced interfacial polarization and enhanced dispersion of the BTO nanoparticles, the leakage current density $\left({J}_{l}\right)$ of the PDMS/BTO@PDA composite during electrical poling is considerably decreased by the formation of the PDA layer, as shown in Fig. 5c.

**Figure 6:**
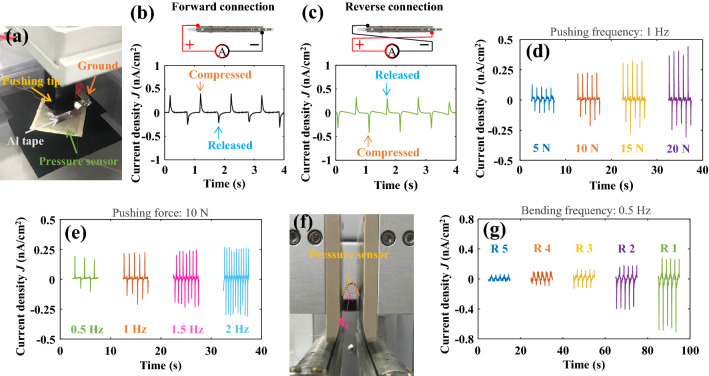
Evaluation of the piezoelectric pressure sensor with the CS NF network electrodes. (**a**) Image of the pressure sensor under the pushing tester (the grounded Al tape is attached to the top surface of the pressure sensor). *J* of the pressure sensor was measured during the pushing study in the (**b**) forward and (**c**) reverse connection modes. *J* of the pressure sensor was measured under various pushing (**d**) forces (5–20 N) and (**e**) frequencies (0.5–2 Hz). (**f**) Image of the pressure sensor under the bending tester (${R}_{\mathrm{c}}$ indicates the radius of curvature). (**g**) *J* of the pressure sensor was measured during the bending study with various radii of curvature ($R_{{\text{c}}} =$ 1–5 mm).

The performance of the piezoelectric pressure sensor with the CS NF network electrodes was evaluated via repetitive pushing and bending studies. In the pushing study (Fig. 6a), the grounded Al tape is attached to the top surface of the pressure sensor to prevent the effect of triboelectricity on the short-circuit current $\left({I}_{\mathrm{sc}}\right)$ signal^66^. Figure 6b shows the current density $J \left(={I}_{\mathrm{sc}}/A\right)$ measured under a pushing force of 20 N at a frequency of 1 Hz. During compression and release of the pushing tip, alternating positive and negative current peaks are produced via the generated piezoelectric potential within the PDMS/BTO@PDA composite^67^. To validate the origin of the observed current signals, a switching polarity test was performed^68,69^. ${I}_{\mathrm{sc}}$ was measured in the forward (Fig. 6b) and reverse connection modes (Fig. 6c). In the reverse connection mode, *J* exhibits approximately the same amplitudes as those observed in the forward connection mode, with the polarities of the signals reversed. Hence, the measured ${I}_{\mathrm{sc}}$ is mainly due to the piezoelectric effect, and the current contributed by other possible artifacts is negligible. Subsequently, the piezoelectric sensor was subjected to basic performance studies. First, *J* was measured by gradually increasing the pushing force. As shown in Fig. 6d, the peak-to-peak current density (*J*_pp_) steadily increases from 0.20 to 0.63 $\mathrm{nA}/{\mathrm{cm}}^{2}$ with an increase in pushing force from 5 to 20 N. The relationship between *J*_pp_ and pushing force is approximately linear, with a sensitivity of 0.029 ${\mathrm{nA}}/{\mathrm{cm}}^{2}\cdot {\mathrm{N}}$ Additionally, the effect of pushing frequency on sensing performance was studied. Figure 6e shows the steady increase of *J*_pp_ from 0.27 $\mathrm{nA}/{\mathrm{cm}}^{2}$ to 0.53 $\mathrm{nA}/{\mathrm{cm}}^{2}$ with an increase in pushing frequency from 0.5 to 2.0 Hz. This relationship between the strain rate and ${I}_{\mathrm{sc}}$ is a representative feature of piezoelectric devices, which is well elaborated on in the literature^70,71^.

We also performed bending studies to evaluate the flexibilities of the pressure sensors. As shown in Fig. 6f, the bending radius is precisely controlled to monitor its effect on *J*. As shown in Fig. 6g, the current signals comprise alternating positive and negative peaks corresponding to the bending and releasing motions of the pressure sensor. As the radius of curvature $\left({R}_{\mathrm{c}}\right)$ decreases from 5 to 3 mm, *J*_pp_ slowly increases from 0.09 to 0.24 $\mathrm{nA}/{\mathrm{cm}}^{2}$. However, when ${R}_{\mathrm{c}}$ is reduced to < 3 mm, *J*_pp_ increases sharply to 0.57 $\mathrm{nA}/{\mathrm{cm}}^{2}$ $\left({R}_{\mathrm{c}}=2 \mathrm{mm}\right)$ and 0.94 $\mathrm{nA}/{\mathrm{cm}}^{2}$ $\left( {R_{{\text{c}}} = 1 {\text{mm}}} \right)$. When the pressure sensor is strained via bending, a high piezoelectric potential is formed within the PDMS/BTO@PDA composite, forcing electrons to flow through the external electric circuit. Thus, with a decrease in the bending radius, the strain in the piezoelectric composite intensifies, resulting in an enhanced piezoelectric potential and *J*_pp_. The evaluation results of the piezoelectric pressure sensor indicate that the highly uniform CS NF network was effective to conduct electrical poling for the PDMS/BTO@PDA composite. At the same time, the CS NF network was an effective electrode material for the flexible piezoelectric pressure sensor under mechanical stimuli.

## Conclusions

In this study, the topology of the PVDF-Pt CS NF network was engineered to enhance its performance as an FTE. Three distinct topologies of PVDF nanofiber networks were prepared using circular, square, and rectangular electrode collectors during electrospinning. While the CS NF network prepared using the circular electrode collector exhibited an almost isotropic ${R}_{\mathrm{s}}$ owing to its randomly aligned nanofibers, those obtained using the square and rectangular electrode collectors displayed anisotropic ${R}_{\mathrm{s}}$ values. The smallest ${R}_{\mathrm{s}}$ was observed using the CS NF network prepared with the square electrode collector, followed by those of the networks prepared using the circular and rectangular electrode collectors. In contrast, the highest *T* was observed using the CS NF network prepared using the rectangular electrode collector, followed by those of the networks prepared using the circular and square electrode collectors. The effect of nanofiber alignment on ${R}_{\mathrm{s}}$ and *T* was explained using node theory. The actual node density differed from the ideal node density when the network was prepared using the circular electrode collector because of the nanofiber bundles formed. Among the three CS NF networks, that prepared using the circular electrode collector exhibited the largest $\Pi \left(330.5\right)$ and smallest $n\left(0.026\right)$, as it simultaneously displayed a considerably small ${R}_{\mathrm{s}}$ and high *T* (e.g., in the CS NF network electrospun for 3 min, $R_{{\text{s}}} = 11.29\; {\Omega }/{\text{sq}}.$ and $T = 83.48{\text{\% }}$ at 550 nm). Meanwhile, the CS NF network obtained using the rectangular electrode collector exhibited superior flexibilities in certain strain directions compared to those of the networks prepared using the circular and square electrode collectors. This suggested that the flexibility depended on the Kirigami design of the nanofiber network. In randomly aligned nanofiber networks, several nanofibers were always closely aligned with the directions of strain induced by bending and stretching, resulting in the destruction of the nanofibers. However, in the CS NF network prepared using the rectangular electrode collector, the strain was accommodated by the rearrangement of the nanofibers. Piezoelectric pressure sensors based on PDMS/BTO@PDA piezoelectric layers were fabricated using the prepared CS NF networks as the top and bottom electrodes. In this process, the CS NF networks were used as the conductive materials during the electrical poling process of the piezoelectric composite layer. The highly uniform nanofiber network was advantageous to conduct electrical poling for the piezoelectric composite layer. The dependencies of ${I}_{\mathrm{sc}}$ on the pushing force and frequency, and bending radius, were evaluated via pushing and bending studies. This study reveals the competitiveness of the PVDF-Pt CS NF network as an electrode for use in flexible electronic devices.

## Supplementary Information

Supplementary Information.

## Data Availability

The datasets generated during and/or analysed during the current study are available from the corresponding author on reasonable request.
