# Supplementary material for: Engineered kirigami design of PVDF-Pt core–shell nanofiber network for flexible transparent electrode
Source: Sci Rep. 2023 Feb 14;13:2582. doi: 10.1038/s41598-023-29812-5 (PMC9929047; doi:10.1038/s41598-023-29812-5)
Supplement: Supplementary file 1 — Supplementary Information. [file 41598_2023_29812_MOESM1_ESM.pdf]

Supplementary Information

Engineered kirigami design of PVDF-Pt core-shell nanofiber network for flexible transparent electrode

Heesung Park [1], Hyeokjun Si [1], Junseo Gu [1], Donghyun Lee [1], Donghyuck Park [1], Young-In Lee [2], Kwanlae Kim [1,]*

[1] Department of Manufacturing Systems and Design Engineering (MSDE), Seoul National University of Science and Technology (SeoulTech), Seoul 01811, Republic of Korea

[2] Department of Materials Science and Engineering, Seoul National University of Science and Technology (Seoultech), Seoul 01811, Republic of Korea

[*]Author to whom correspondence should be addressed: klkim@seoultech.ac.kr

Experimental procedure

Fabrication of the $BaTiO_3$ (BTO) nanoparticles functionalized with polydopamine (PDA)

BTO nanoparticles (5 g with diameters of 200 nm, US Research Nanomaterials, Houston, TX, USA) were dispersed in 30 mL H_2O_2 solution (30 wt.% in H_2O, Daejung Chemicals, Siheung, Republic of Korea) via ultrasonication. The dispersed nanoparticles were hydroxylated via heating at 104 ℃ for 4 h. Subsequently, they were centrifuged at 4000 rpm for 5 min, washed several times using deionized (DI) H_2O, and dried at 60 ℃ for 12 h to yield BTO-OH nanoparticles. These nanoparticles (2 g) were ultrasonically dispersed in a Tris-HCl solution (10 mM, pH 8.5, 99 %, Daejung Chemicals) for 30 min. Subsequently, 0.6 g of dopamine hydrochloride ($C_8H_{11}NO_2 \cdot HCl$, 99 %, Alfa Aesar, Haverhill, MA, USA) was added to the solution, which was then stirred at room temperature for 24 h. The resultant product was centrifuged at 4000 rpm for 10 min and washed several times with DI water. Finally, BTO nanoparticles functionalized with PDA (BTO@PDA) were obtained after drying at 60 ℃ for 12 h [1,2].

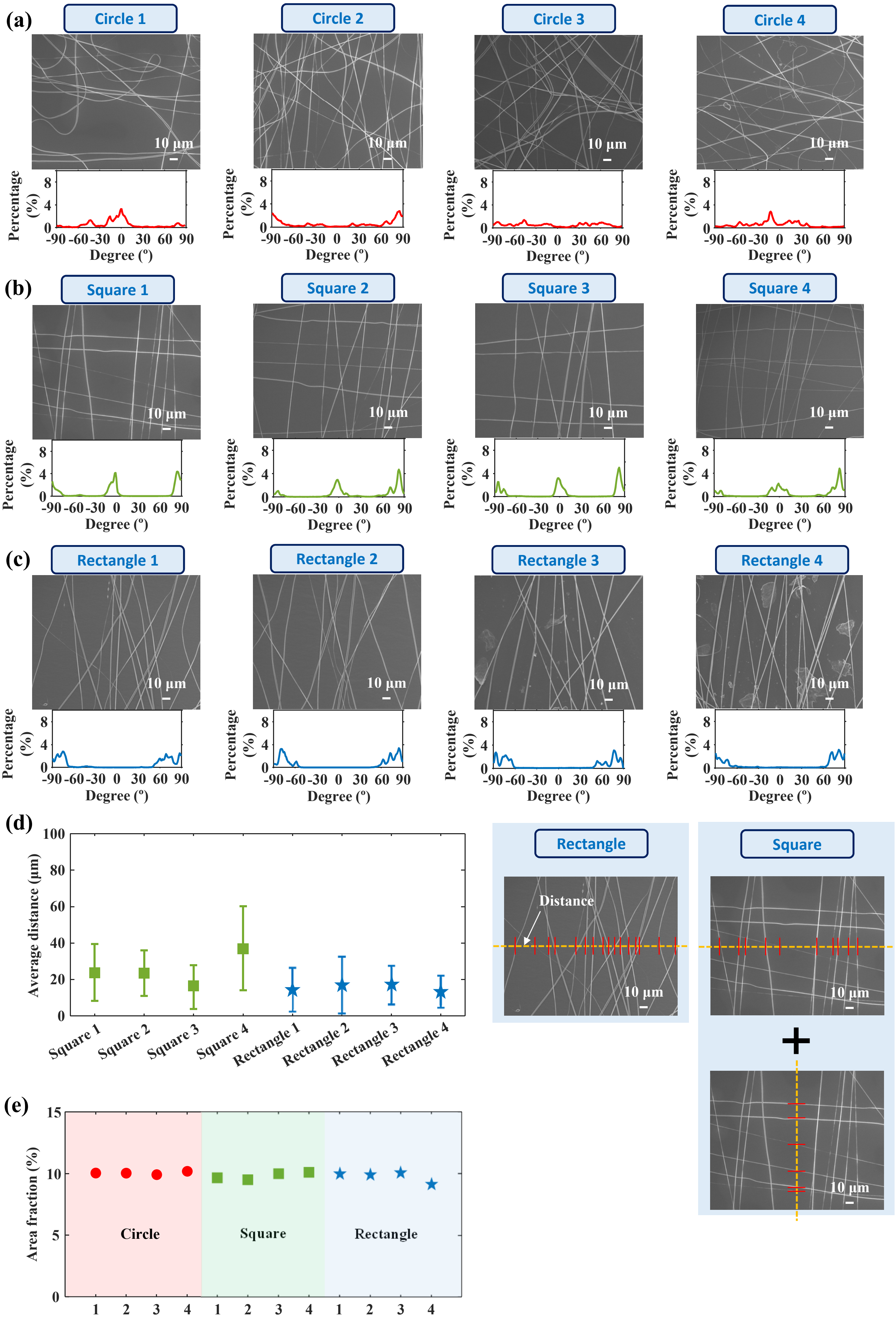

(a)
Circle 1
Circle 2
Circle 3
Circle 4
10 μm
Percentage (%)
Degree (°)
(b)
Square 1
Square 2
Square 3
Square 4
(c)
Rectangle 1
Rectangle 2
Rectangle 3
Rectangle 4
(d)
Average distance (μm)
Rectangle
Square
Distance
(e)
Area fraction (%)
Circle
Square
Rectangle

Fig. S1. Analysis of PVDF nanofiber alignments. SEM images of the nanofiber networks fabricated using (a) circular, (b) square, and (c) rectangular electrode collectors. These twelve images were taken from the twelve distinct samples of nanofiber networks produced under the same electrospinning condition (1 minute). (d) Average distance between two neighboring nanofibers in the CS NF network from the square and rectangular electrode collectors. The distance values in "rectangle" image were obtained by measuring the distances between the intersecting points between nanofibers and the lateral dotted line. In "square" image, the same methodology was applied to both vertical and lateral directions. The average distances in (d) were measured using the eight images in (b) and (c). As the nanofibers in (a) are randomly aligned, average distances between nanofibers were not measured. (e) Area fraction of nanofibers for each image in (a) – (c). Area fraction was computed using ImageJ software [3,4].

Figs. S1(a)–(c) show that unlike the nanofiber networks from the circular electrode collector, those from the square and rectangular electrode collectors clearly exhibit alignments. However, the structure of nanofiber network was not precisely controlled by the present fabrication process. Fig. S1(d) shows that the average distance between neighboring nanofibers varied depending on samples even through they were produced from the same type of electrode collector. The area fractions of nanofibers (Fig. S1(e)) were also slightly different depending on samples. Overall, even though nanofiber networks were produced from the same electrode collector, the topology of nanofiber network slightly varied from sample to sample. Nevertheless, for the three types of electrode collectors the topology models of nanofiber networks (shown in Fig. 2(a)) were developed based on the SEM images and the statistical analysis results to highlight the clearly different topologies among the nanofiber networks.

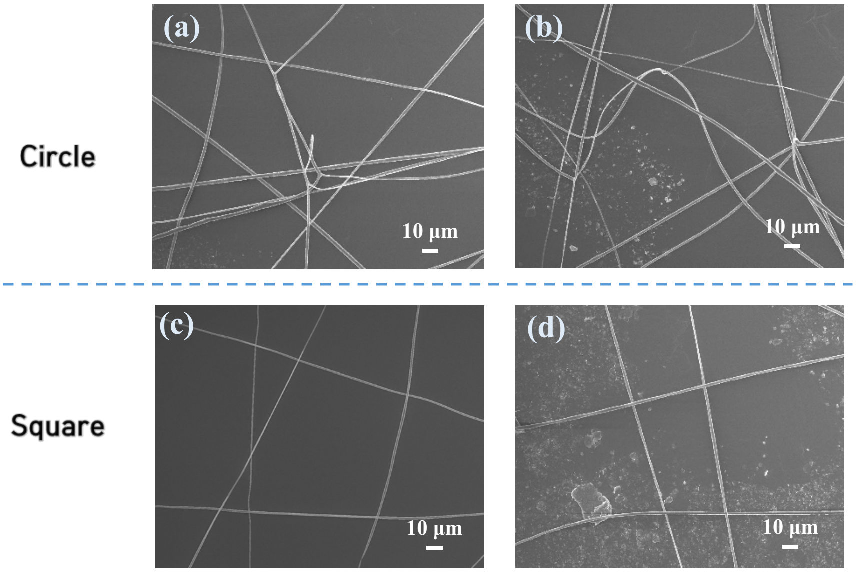

Fig. S2. SEM images of the PVDF nanofibers obtained using the (a), (b) circular and (c), (d) square electrode collectors.

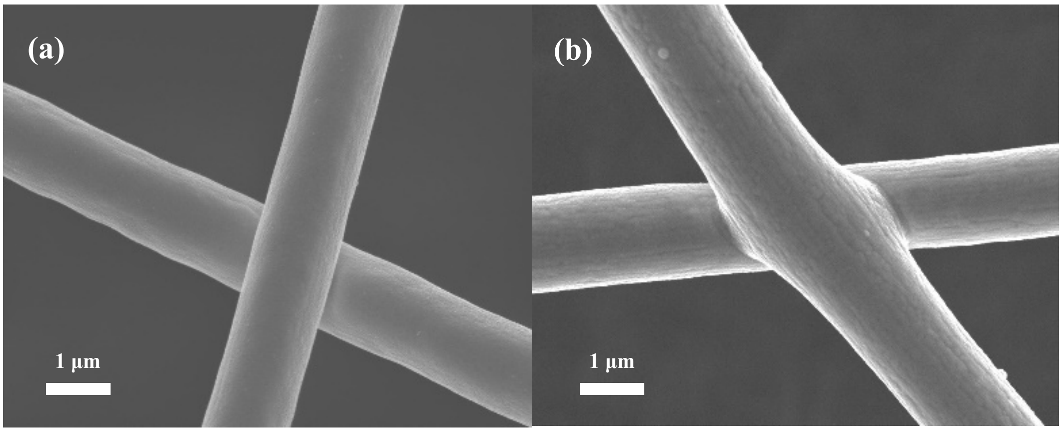

Fig. S3. SEM images of the PVDF nanofibers (a) before and (b) after Pt sputtering for 6 min. In (b), the junction of the PVDF nanofibers is fused by the Pt layers.

(a)

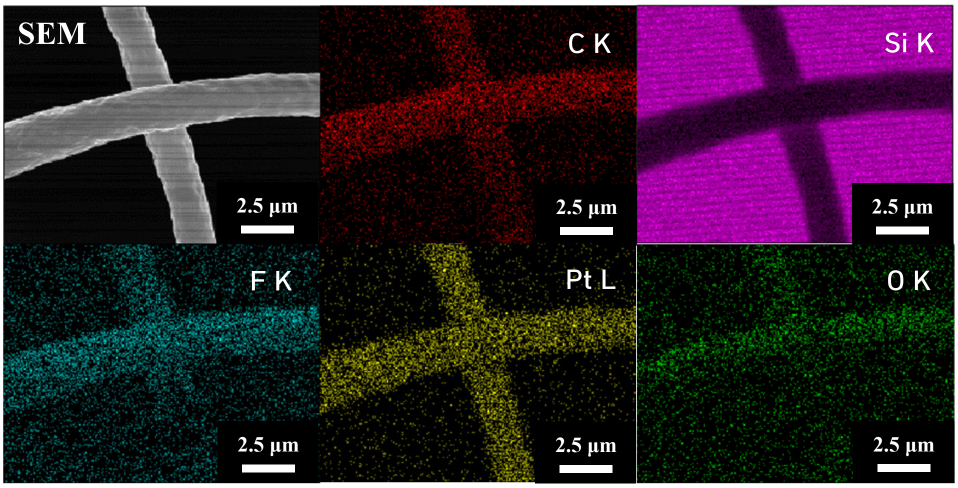

(b)

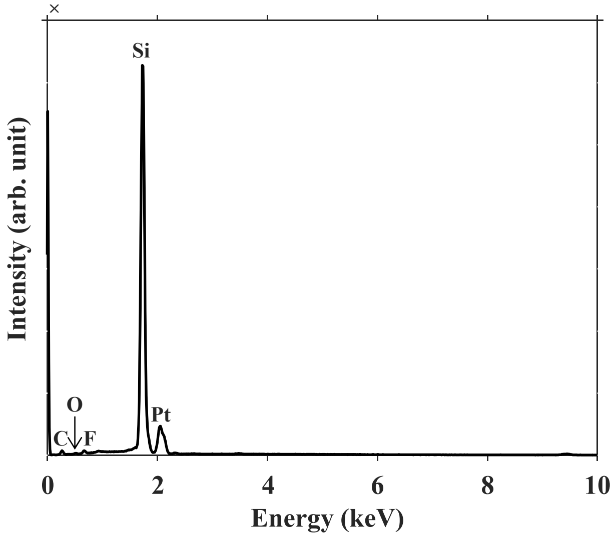

Fig. S4. (a) SEM image and EDS elemental maps of a CS NF network, and the (b) ED spectrum. Notably, the CS NF network was transferred to a Si wafer substrate for use in EDS, and thus, a large amount of Si is detected.

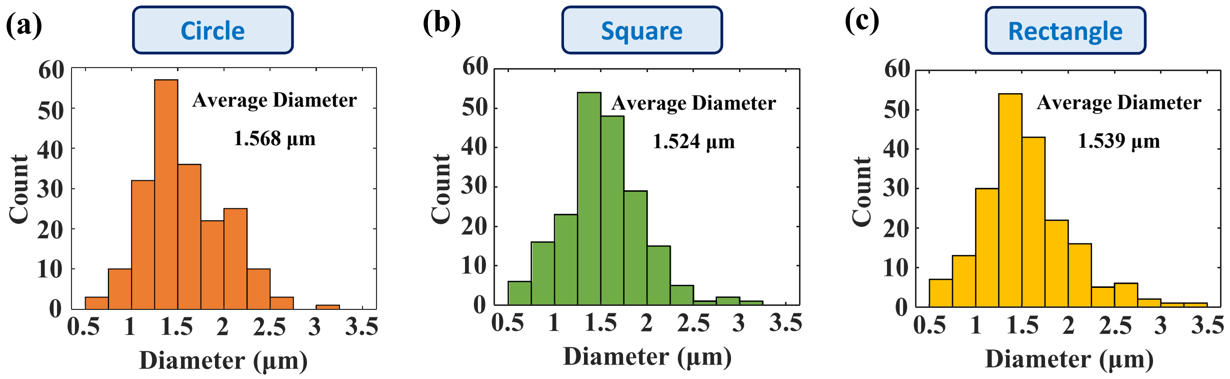

Fig. S5. Histograms of the diameters of the PVDF nanofibers produced using the (a) circular, (b) square, and (c) rectangular electrode collectors. The diameters were measured using the SEM images.

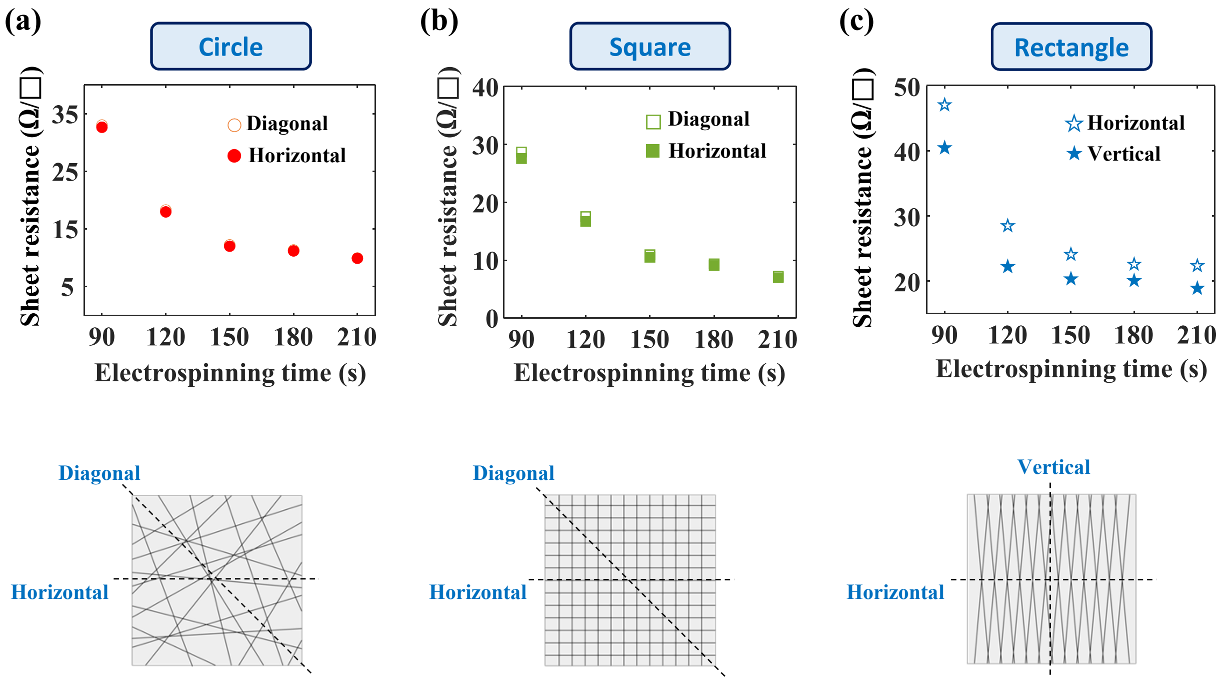

Fig. S6. Sheet resistances (R_s) of the CS NF networks with respect to electrospinning time. The R_s values of the CS NF networks obtained using the (a) circular, (b) square, and (c) rectangular electrode collectors were measured along the directions indicated in the schematic diagrams.

In Fig. S6(a), there is no noticeable difference in R_s according to the measurement direction. This is due to the randomly aligned PVDF nanofibers, as shown in Fig. S1(a). In Fig. S6(b), the effect of the measurement direction on R_s is also very small, but R_s measured in the horizontal direction is noticeably smaller than that measured in the diagonal direction when the electrospinning time is 90–120 s. In Fig. S6(c), R_s measured in the vertical direction is much smaller than that measured in the horizontal direction, even when the electrospinning time is increased to 210 s.

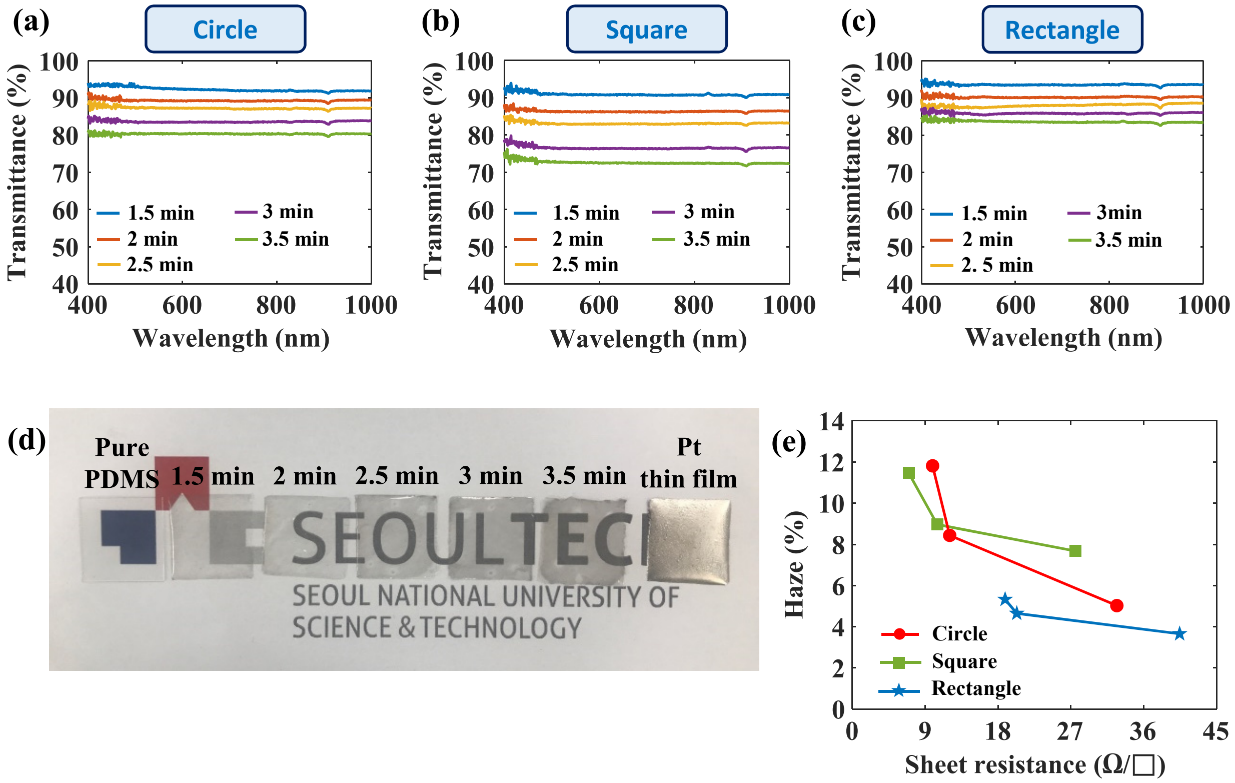

Fig. S7. Transparencies of the CS NF networks. Transmittances of the CS NF networks prepared using the (a) circular, (b) square, or (c) rectangular electrode collector with respect to various wavelengths (400–1000 nm). (d) Images of the CS NF networks fabricated using various electrospinning times (1.5–3.5 min). (e) Haze values with respect to R_s of the CS NF networks fabricated using 1.5, 2.5, and 3.5 min electrospinning times.

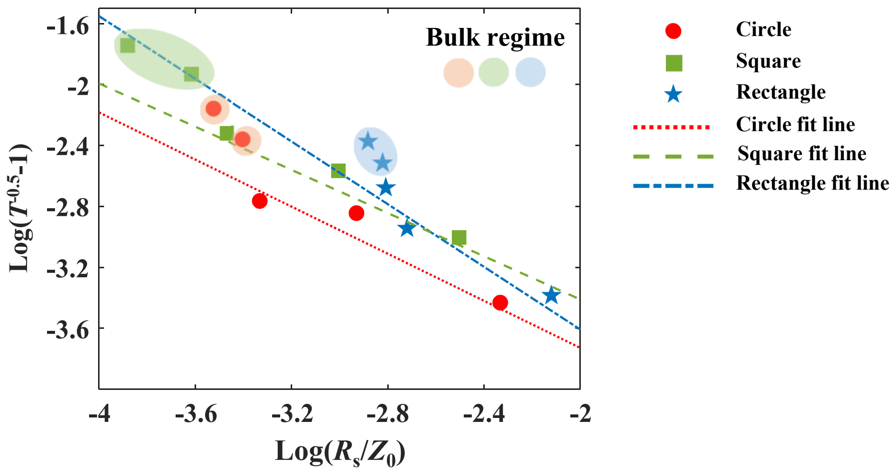

Fig. S8. Plots of $\mathrm{Log}(T^{-0.5}-1)$ as a function of $\mathrm{Log}(R_s/Z_0)$ for the CS NF networks.

According to Eq. (1), linear relationships between $\mathrm{Log}(T^{-0.5}-1)$ and $\mathrm{Log}(R_s/Z_0)$ may be observed in nanostructured transparent conductors in the percolation regime. Accordingly, the fit lines of the CS NF networks prepared using the circular, square, and rectangular electrode collectors are shown in Fig. S8. The CS NF networks in the bulk regime are indicated by shaded regions.

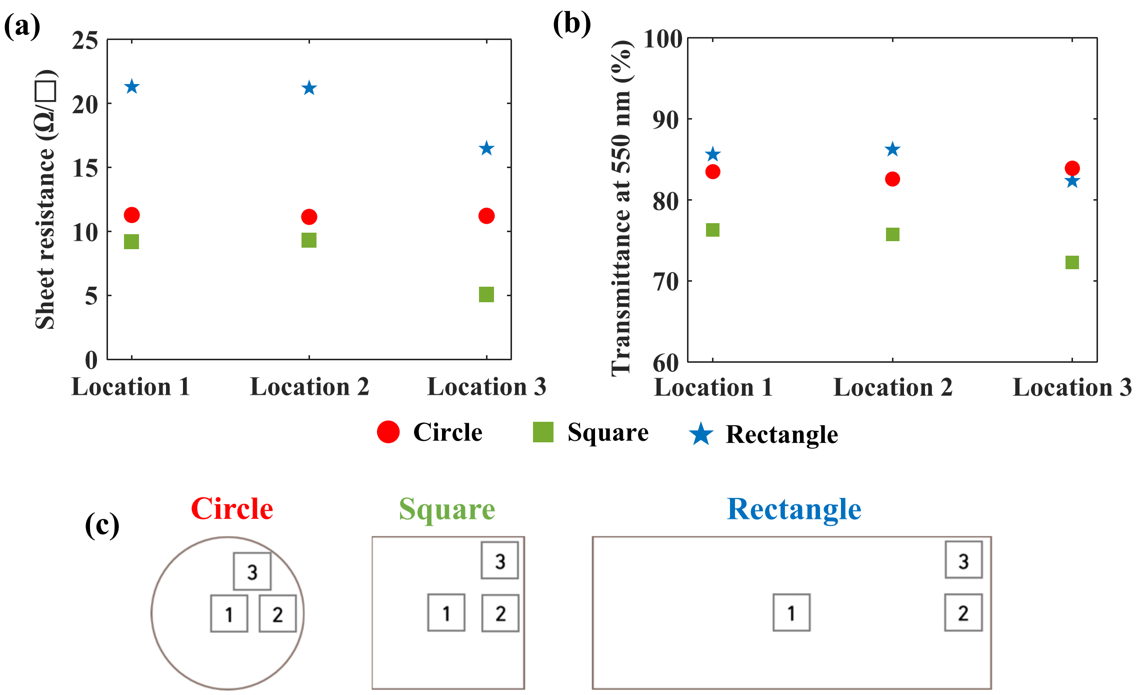

Fig. S9. Dependencies of R_s and T on sampling locations. (a) R_s, and (b) T of the CS NF networks from the circular, square, and rectangular electrode collectors. The location numbers in (a) and (b) indicate that R_s and T measurements were conducted for the CS NF network samples taken from the locations shown in (c). The CS NF networks were fabricated under the same condition (an electrospinning time of 3 min, and a sputtering time of 6 min).

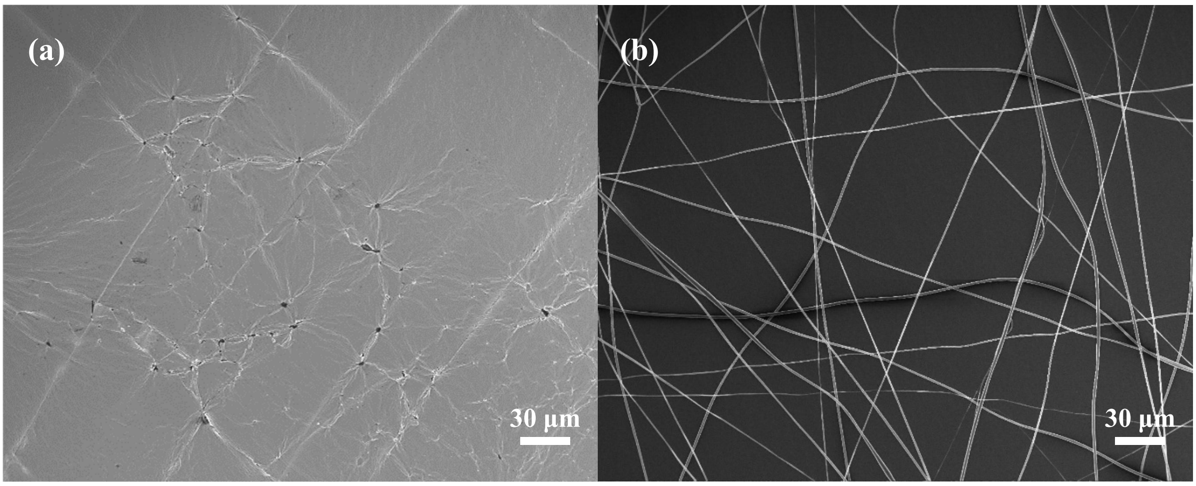

Fig. S10. Mechanical durabilities of a Pt thin film and PVDF-Pt CS NF network. SEM images of a (a) Pt thin film and (b) CS NF network after 1000 cycles of bending and releasing at a bending radius of 1 mm. PDMS was used as a substrate in the bending study, and the Pt thin film was deposited via sputtering for 6 min. The CS NF network was fabricated using a circular electrode collector via electrospinning for 180 s and sputtering for 6 min.

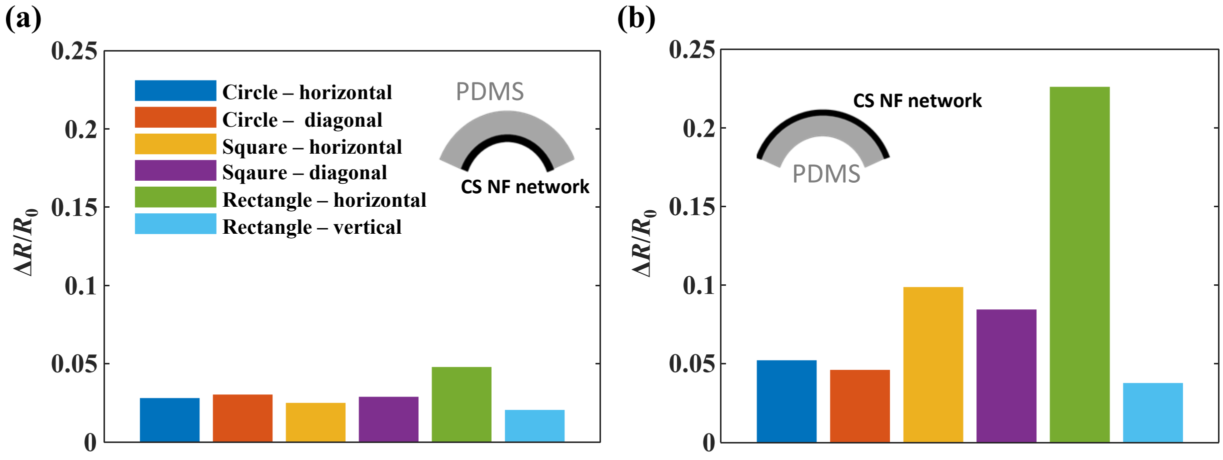

Fig. S11. $\Delta R/R_0$ values of the CS NF networks after 1000 cycles of bending and releasing. In (a), the CS NF network was placed at the inner surface of the bent PDMS substrate to induce compressive stress. In (b), the CS NF network was positioned at the outer surface of the bent PDMS substrate to induce tensile stress. In both studies, bending was performed at a bending radius of 1 mm.

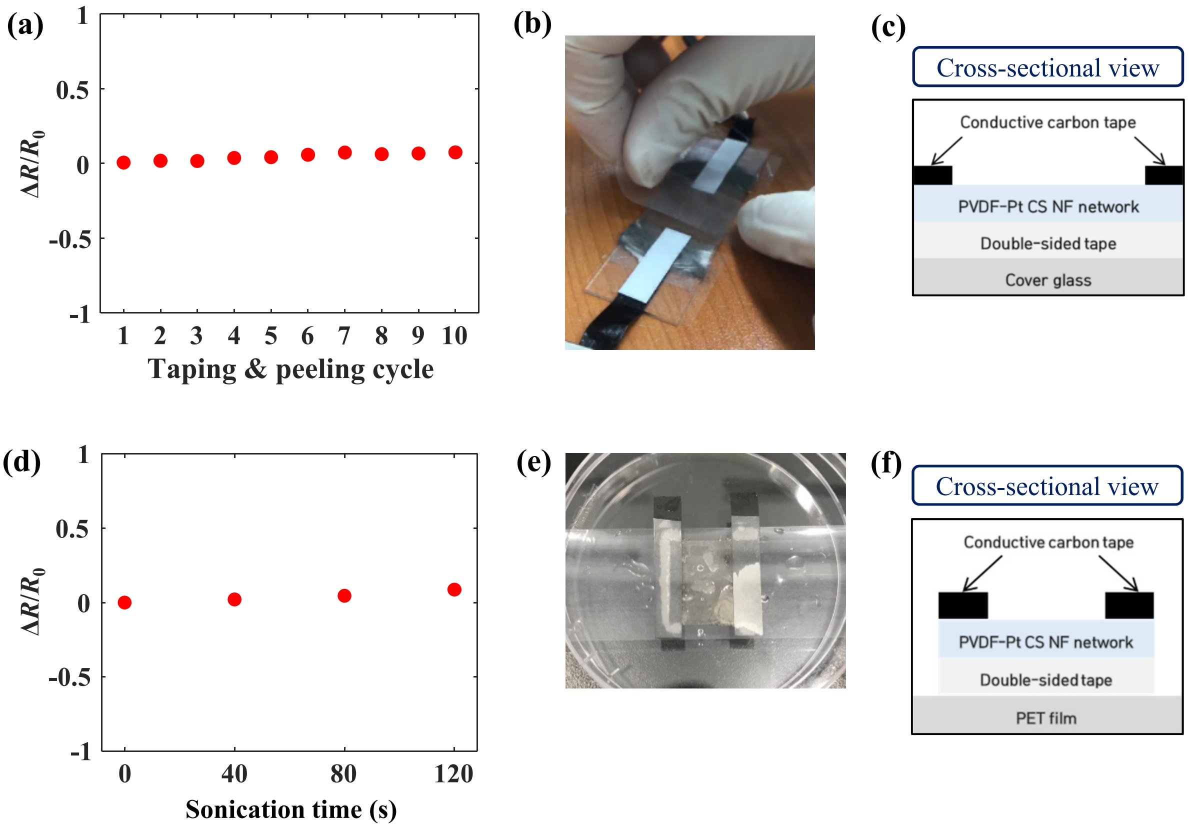

Fig. S12. Durability evaluation of the CS NF networks obtained using the circular electrode collector under harsh conditions. (a) $\Delta R/R_0$ values of the CS NF network with an increase in the cycles of taping and peeling, (b) photography of taping test, (c) schematic cross-sectional view of the CS NF network attached to the cover glass, (d) $\Delta R/R_0$ values of the CS NF network with respect to sonication time, (e) photography of sonication test, and (f) schematic cross-sectional view of the CS NF network attached to the PET film.

To assess the durability of CS NF networks under harsh conditions, $\Delta R/R_0$ was measured under 10 cycles of taping and peeling. A 3M tape (Scotch Crystal Clear Tape) was attached and peeled off by hand as Fig. S12(b) shows [5]. Meanwhile, the sonication test was carried out by soaking a CS NF network in DI water for 120 s. An ultrasonic bath (CPX1800H-E, Branson, U.S.A.) was used to perform the sonication test at a frequency of 40 kHz [6]. Note that in this durability test, the resistance measurement method was the same with the one used in the bending test in Fig. 4. In the durability tests, the CS NF network from the circular electrode collector was used.

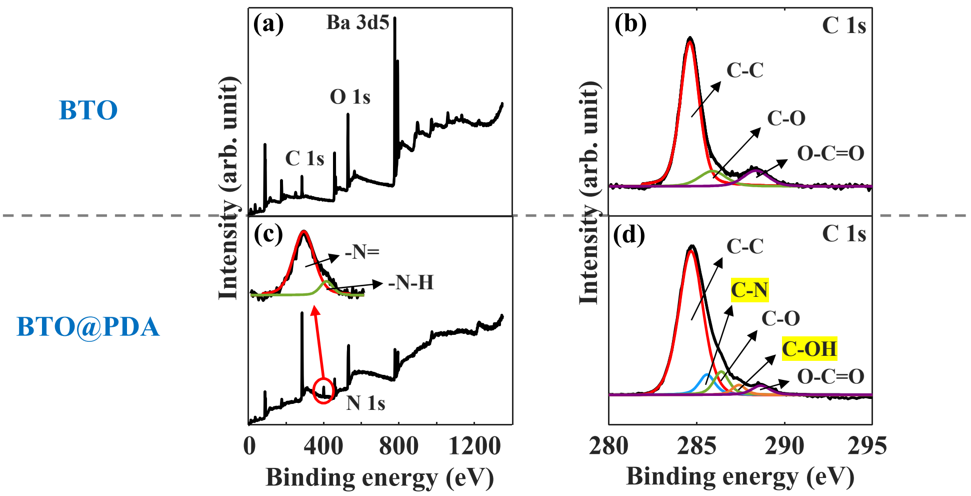

Fig. S13. XP spectra of BTO and BTO@PDA nanoparticles. (a) XPS wide scan and (b) C 1s core-level spectra of BTO, and (c) XPS wide scan (including N 1s core-level) and (d) C 1s core-level spectra of the BTO@PDA nanoparticles.

The PDA layers on the BTO nanoparticles may be indicated by the appearance of the new peak at a binding energy of ~400 eV due to the imine (–N=) and amine (–N–H) species. Additionally, when the C 1s core-level spectrum is deconvoluted, two new components (C–N and C–OH species) are identified, indicating the presence of the PDA layer [7,8].

References

1. Jia, N. *et al*. Enhanced β-crystalline phase in poly(vinylidene fluoride) films by polydopamine-coated BaTiO3 nanoparticles. *Mater. Lett.* **139**, 212–215 (2015).
2. Hanani, Z. *et al*. Lead-free nanocomposite piezoelectric nanogenerator film for biomechanical energy harvesting. *Nano Energy* **81**, 105661 (2021).
3. Stachewicz, U., Modaresifar, F., Bailey, R. J., Peijs, T. & Barber, A. H. Manufacture of void-free electrospun polymer nanofiber composites with optimized mechanical properties. *ACS Appl. Mater. Interfaces* **4**, 2577–2582 (2012).
4. Ravi, S. K. *et al*. Hydro-assisted self-regenerating brominated *N*-alkylated thiophene diketopyrrolopyrrole dye nanofibers—a sustainable synthesis route for renewable air filter materials. *Small* **16**, 1906319 (2020).
5. He, T., Xie, A., Reneker, D. H. & Zhu, Y. A tough and high-performance transparent electrode from a scalable and transfer-free method. *ACS nano* **8**, 4782–4789 (2014).
6. Kwon, Y. B., Kim, J. H. & Kim, Y. K. Efficient protection of silver nanowire transparent electrodes by all-biorenewable layer-by-layer assembled thin films. *ACS Appl. Mater. Interfaces* 1**4**, 25993–26003 (2022).
7. Yang, D. *et al*. Dopamine and silane functionalized barium titanate with improved electromechanical properties for silicone dielectric elastomers. *RSC Adv.* **6**, 90172–90183 (2016).
8. Zhang, W., Kai, Y., Lin, J., Huang, Y. & Liu, X. Enhancing dielectric and mechanical properties of poly(arylene ether nitrile) based composites by introducing low content "core-shell" like structured MXene&PDA@ $BaTiO_3$. *High Perform. Polym.* **33**, 1–13 (2021).
